# The Nexus Between Periodontal Inflammation and Dysbiosis

**DOI:** 10.3389/fimmu.2020.00511

**Published:** 2020-03-31

**Authors:** Thomas E. Van Dyke, P. Mark Bartold, Eric C. Reynolds

**Affiliations:** ^1^The Forsyth Institute, Cambridge, MA, United States; ^2^School of Dentistry, University of Adelaide, Adelaide, SA, Australia; ^3^Melbourne Dental School, The University of Melbourne, Melbourne, VIC, Australia

**Keywords:** periodontal inflammation, periodontal infection, periodontal pathogen, gingivitis, periodontitis

## Abstract

The nexus between periodontal inflammation and the polymicrobial biofilm in the gingival sulcus is critical to understanding the pathobiology of periodontitis. Both play a major role in the etiology and pathogenesis of periodontal diseases and each reinforces the other. However, this nexus is also at the center of a significant conundrum for periodontology. For all mucosal polymicrobial biofilms, the most confounding issue is the paradoxical relationship between inflammation, infection, and disease. Despite significant advances made in both periodontal microbiology and periodontal pathobiology, the issue of which comes first, the inflammatory response or the change to a dysbiotic subgingival microbiota, is still debated. In this paper, we present a model for the pathogenesis of periodontitis based on the central role of inflammation and how this modulates the polymicrobial biofilm within the context of the continuum of health, gingivitis, and periodontitis. We propose a new model termed “Inflammation-Mediated Polymicrobial-Emergence and Dysbiotic-Exacerbation” (IMPEDE), which is designed to integrate into and complement the 2017 World Workshop Classification of Periodontitis.

## Introduction

Periodontitis is currently considered by the American Academy of Periodontology to be an inflammatory disease initiated by bacteria (1). While this represented a paradigm shift relating to the pathogenesis of periodontitis at the time, it still remains unclear how this relationship plays out during the development of periodontitis. Clearly, one cannot be there without the other. The confounding issue is the relationship between inflammation and disease; which comes first, the immune response or the change in the homeostatic integrity of the mucosal polymicrobial biofilm (2). While remarkable progress has been made in the last 10 years in both the microbiology and immunology of periodontitis, this relationship has not been clarified.

A number of questions remain to be investigated and answered. What drives the shift from the localized and contained inflammatory response of gingivitis to progressive, destructive periodontitis? When and how does the subgingival microbiome become dysbiotic? Is it a spontaneous evolution of maturing biofilms or is it driven by the changing environment mediated by the host response? What is the temporal relationship between the dysbiotic microbiome and the innate and acquired immune response? Is bacterial invasion of tissues an initiator, or consequence, of disease?

In this review, we will critically examine the evidence that addresses these questions in an effort to better understand the interface between inflammation and dysbiosis. Our understanding of the initiation of periodontitis requires reassessment. Management of disease by attempted removal of bacteria (debridement) is only partially effective for periodontitis and fails in high-risk individuals. Detailed consideration of the host response, genetic, and environmental factors as well as the microbiology are required. The identification of a true infectious pathogen for periodontitis has eluded researchers for decades. More recently, concepts focused on controlling the inflammation to control the polymicrobial biofilm dysbiosis have emerged for periodontitis as well as a number of other polymicrobial biofilm-based-inflammatory diseases (3–7).

## Pathology of Periodontitis

The critical questions to understanding any pathological process are (1) What initiates disease, (2) What exacerbates disease, and (3) What resolves disease? The context here is periodontal disease:

### Initiation

Low grade “surveillance” inflammation; that is, the presence of neutrophils in gingiva with no clinical signs of inflammation, appears to be normal homeostasis in humans. In most people, failure to disrupt the polymicrobial biofilm (plaque) accumulation at the gingival margin of teeth on a regular basis will lead to gingivitis, a destructive inflammatory lesion resulting in loss of collagen locally that is reversible upon resolution of the inflammation. Gingivitis is a chronic inflammatory lesion that in certain individuals can become destructive periodontitis. Periodontitis is distinguished from gingivitis by the destruction of supporting periodontal tissues, including periodontal ligament and alveolar bone, that is irreversible upon removal of the bacterial challenge (8). The stimulus for progression from a stable chronic gingivitis to destructive periodontitis remains elusive. To date, no specific bacterial species or antigens have been demonstrated within the tissues at this stage in the pathogenesis of periodontitis (9). Instead the initiation of destructive periodontitis has been associated with dysbiosis where the diversity, richness, and relative proportions of species in the subgingival microbiota are altered (10).

### Exacerbation

We are defining this as the switch from gingivitis to periodontitis. Here, the data are limited to associations and therefore, cause and effect inferences are limited. The observations are consistent with a changing subgingival microenvironment related to chronic inflammation with a change in the composition and proportions of the bacterial species of the polymicrobial biofilm that characterizes dysbiosis. With time, there is an acquired immune response that can be either destructive or reparative in different circumstances (11). There is reasonable evidence in other systems that inflammation can cause dysbiosis (4–7), and there is reasonable evidence that dysbiosis induces inflammation (12). It is highly unlikely that either the bacteria or the host response is the sole driver of disease. However, the exact temporal relationship and contribution of each is not well-described.

### Resolution

Resolution in this context refers to resolution of disease. This encompasses resolution of inflammation and return of tissue homeostasis and the re-establishment of a truly commensal plaque microbiome that is also in a homeostatic relationship with the host. This does not appear to occur spontaneously due to the heavy bacterial load that has increased species diversity that seems to be driven by the inflammatory and periodontal pocket environment. The dysbiotic subgingival plaque microbiome drives further inflammation perpetuating disease. Clinically, we have learned that this can be reversed by reduction in bacterial load that leads to reduced inflammation, or by reduction of inflammation, which prevents tissue destruction and modifies dysbiosis (13). Furthermore, this concept is supported by animal studies of experimental periodontitis where anti-inflammatory therapies not only inhibited periodontal bone resorption but also decreased the bacterial biomass and reversed dysbiosis (14, 15).

## The Interface Between Inflammation and Dysbiosis

Our understanding of the relationship between inflammation and the polymicrobial biofilm was greatly enhanced by the discovery of the *active* pathways of resolution of inflammation and how these pathways are operative in periodontitis (16). Resolution of inflammation is regulated by Specialized Proresolving Mediators of inflammation (SPMs) comprising low molecular weight eicosanoids derived from arachidonic acid and omega-3 polyunsaturated fatty acids call lipoxins, resolvins protectins, and maresins (17). These molecules are classified as receptor agonists, not inhibitors or antagonists, that naturally resolve the inflammatory response through a feed-forward mechanism after binding to specific receptors on inflammatory cells. Since their actions are receptor mediated, their actions are specific, unlike inhibitors and antagonists. Importantly, discovery of this regulatory system opened the doors for natural manipulation of the inflammatory response in chronic inflammatory diseases. Further, it provided an avenue for the investigation of the impact of inflammation on the microbiome.

### Dysbiosis

Much of the dysbiosis theory in periodontitis is an extension of gut microbiome research (18). The transition in the polymicrobial community from largely gram-positive commensal to a gram-negative enriched inflammogenic community is well-established (19, 20).

### Inflammation and Dysbiosis in Periodontitis

The relationship of the periodontal microbiome to development of periodontitis is complex. The proposal that specific pathogens *initiate* dysbiosis and disease is in question due to the lack of a clear association of any putative keystone pathogen with disease initiation in humans. From microbiome analyses of plaque samples taken from healthy, gingivitis, and early periodontitis sites, we know that the bacteria associated with the initiation of disease are largely commensals and the “putative pathogens or pathobionts” that have been associated with disease at a later stage are very minor components of the biofilm at this early stage (20). The shift to a dysbiotic microflora appears to be in large part a function of excess and persistent inflammation and pocket formation that changes the bacterial growth environment. This was first recognized in the early 1990's as the ecological plaque hypothesis (21). In this hypothesis, the subgingival environment exerts selective pressure changing the specific microbial composition driving the change from health to disease.

The gingival microbiome associated with periodontal health is stable over time in dynamic equilibrium with the host. Gingivitis is a stable inflammatory condition and, in many ways, represents homeostasis. Excess, uncontrolled and chronic inflammation results in irreversible destruction of hard and soft tissues known as periodontitis. Disease-associated bacteria are a very small component of the subgingival microflora in health and increase significantly with the development of periodontal pockets and periodontitis (20, 22). In health (and in gingivitis, which is arguably the normal homeostatic condition), organisms seem to self-regulate by interspecies competition creating *microbial homeostasis*. With excess inflammation and pocket formation, initially by soft tissue swelling, the local environment becomes anaerobic and enriched with tissue breakdown products, plasma proteins and hemoglobin from bleeding that select for anaerobic gram-negative, proteolytic bacteria that use essential amino acids, and hemin as an energy source. Hence, overgrowth of specific subsets or consortia of microbes within the polymicrobial biofilm result from changes in the microenvironment (21, 23). Dysbiosis of the periodontal microbiome is clearly associated with periodontitis; however, whether dysbiosis initiates disease or is a consequence of disease initiation has not been definitively demonstrated, but it is clear that the nexus between inflammation and dysbiosis is critical (10, 24).

Bacteria are undoubtedly the principal cause of gingivitis, but it is the host response to those bacteria that dictates whether disease progresses (8). Overwhelming evidence has accrued to demonstrate that it is uncontrolled host inflammatory and immune responses that largely drive the tissue destruction (10).

### Pro-resolving Mediators and the Microbiome

Molecular tools that directly extract and sequence cloned DNA from communities of microorganisms have opened new paths to our understanding of the relationship between human cells and the microbes that colonize the human body (25). Thousands of previously uncultured and unknown microbes have been identified. The study of the *human microbiome* codifies bacterial associations with inflammatory diseases and conditions including those thought to be sterile events (26). Inflammation clearly has a major impact on the microbiome in chronic inflammatory disease. For instance, pathogens that emerge can cause significant inflammatory dysregulation (27) and upregulation of systemic inflammation, as in obesity and type 2 diabetes, causes dysbiosis of the gut microbiome (28).

## Conversion of a Commensal Microbiota to an Opportunistic Pathogenic Microbiota

An important issue to consider in the pathogenesis of periodontitis is the temporal sequence of microbiome changes to periodontal inflammation. There is dysbiosis of the oral microbiome associated with periodontitis (24), but the interplay between bacteria and inflammation is just beginning to be described (14, 29).

Removal of plaque on the teeth reduces inflammation, but the effect is transient, and inflammation returns. Newer data suggest that susceptibility and pathogenesis of periodontitis is mediated by the host response to bacteria (1). Severe periodontitis is characterized by excess inflammation that includes oxidative stress (30) and exuberant cytokine production (31). Longitudinal studies suggest that inflammation predicts disease progression and overgrowth of pathogens in periodontitis occurs after onset of disease (32).

The SPM RvE1, when used as a topic therapeutic to treat inflammation in periodontitis prevents and reverses disease and promotes bone remodeling (23). A relevant observation is that RvE1 caused spontaneous disappearance of the periodontal pathogen, *Porphyromonas gingivalis*., without mechanical or antimicrobial therapy. Lee and co-workers described the temporal dynamics of inflammation-induced dysbiosis of the periodontal microbiota and the impact of RvE1 in rat experimental periodontitis (14). Global differential gene expression in periodontal tissue was determined in health, periodontitis and periodontitis treated with topical RvE1 along with 16S rDNA sequencing of the associated microbiome. Topical application of RvE1 in rat periodontitis induced significant regeneration of lost periodontal soft tissues and bone. The shifts in the local microbiota induced by inflammation were markedly rescued by RvE1. These results illustrate two biological principles: (1) local environmental conditions impact the composition of the microbiota, and (2) the impact of inflammation on the microbiome is modifiable. These changes are not observed with *inhibition* of inflammation with NSAIDs or other inhibitors of inflammation (33).

Microbiome shifts induced by inflammation go beyond just overgrowth of certain species. Growth conditions also provide an environment that changes the physiology, pathogenicity, and expression of virulence factors of the polymicrobial biofilm community (10). Transcriptomic analyses performed on the microflora associated with progressive disease revealed that virulence factors are upregulated by both pathogens and health associated commensals (34). Taken together, the data suggest that the inflammatory response and the resident microbiome are linked in a bi-directional balance in health and a bi-directional imbalance in disease. These principles are not limited to oral diseases and are seen in sepsis, IBD and other diseases (35).

## Can Site Specificity be Explained by Inflammation Mediated Periodontal Dysbiosis?

**Figure 1:**
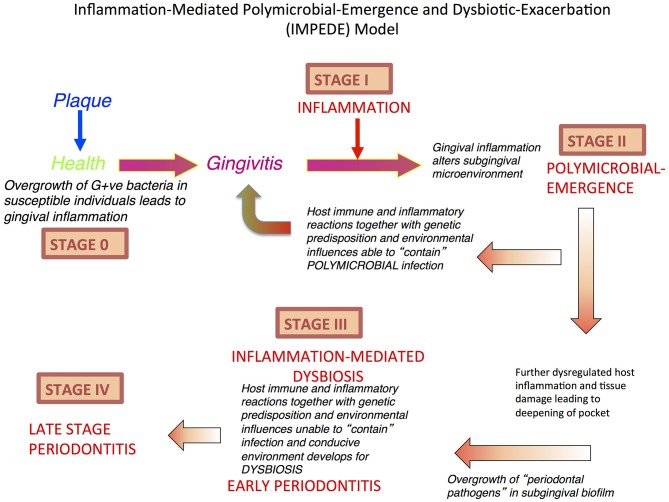
Inflammation-Mediated Polymicrobial Emergence and Dysbiotic Exacerbation (IMPEDE) Model. A proposal to demonstrate how inflammation is a principal driver of plaque-associated periodontitis. This model recognizes 5 stages (0-IV) through which health, gingivitis, and periodontitis may develop, be contained or progress. Stage 0: periodontal health; Stage I: Gingivitis (inflammation); Stage II: Initiation/early periodontitis (Polymicrobial diversity emerges); Stage III: Inflammation mediated dysbiosis and opportunistic infection and Stage IV: Late stage periodontitis.

As early as 1982, it was proposed that the specific microbiota present in deep periodontal pockets may be of significance only at a late stage of periodontal disease. The issue now is: have we made any progress in fully understanding the significance of the temporal sequence of microbial colonization, inflammation, associated tissue damage, subsequent dysbiosis followed by exacerbation of the inflammation-mediated tissue destruction? If this constant interplay between microbes and host inflammatory response is considered as a continuum, or self-sustained feedforward loop (see Figure 1), then it becomes evident that specific bacteria cannot be considered initial causal agents in the pathogenesis of periodontitis (9). More likely, the specific bacteria that have hitherto been identified as being associated with periodontitis appear because of developing disease and as late contributors. The increase in number and volume of specific bacteria is due to the inflammation and pocket formation that provides the anaerobic environment and vital nutrients, hemin, amino acids, and other growth factors via gingival crevicular fluid (36). It is important to understand the temporal and spatial sequence of events in periodontitis and pocket formation, because inflammation always precedes “periodontal pathogen” overgrowth (32). Thus, it seems probable that it is the host response and not the microbes *per se* that determines the eventual outcome of host parasite interactions within periodontal pockets (37). Unraveling how inflammation may drive this process is very important and it is noteworthy that induction of inflammation distant to the periodontium can induce periodontal change.

With regards to pocket formation and dysbiosis, we contend that this occurs only after the initiation of chronic inflammation. The chronic inflammation allows the development of a periodontal pocket that changes the redox and nutrient environment, which increases diversity and species richness of the polymicrobial biofilm. The result is dysbiosis, which reinforces and exacerbates inflammation to initiate bone resorption. Notwithstanding this development, progression at this site is still driven by host inflammation, but now it is exacerbated by, and associated with, a dysbiotic polymicrobial-host dysregulated inflammation. Importantly, even at this stage, if inflammation can be resolved (as discussed above), then the commensal species may be able to antagonize the pathobionts such that oral homeostasis (eubiosis) can return to a treated site after debridement of the dysbiotic biofilm (23). This concept is predicated on the understanding that even with the emergence and eventual establishment of a dysbiotic subgingival polymicrobial biofilm, the commensal microbes have not been eliminated, but merely repressed until such time that the environment allows their return as the principal polymicrobial biofilm components compatible with health (38).

## Unifying Concept

**Figure 2:**
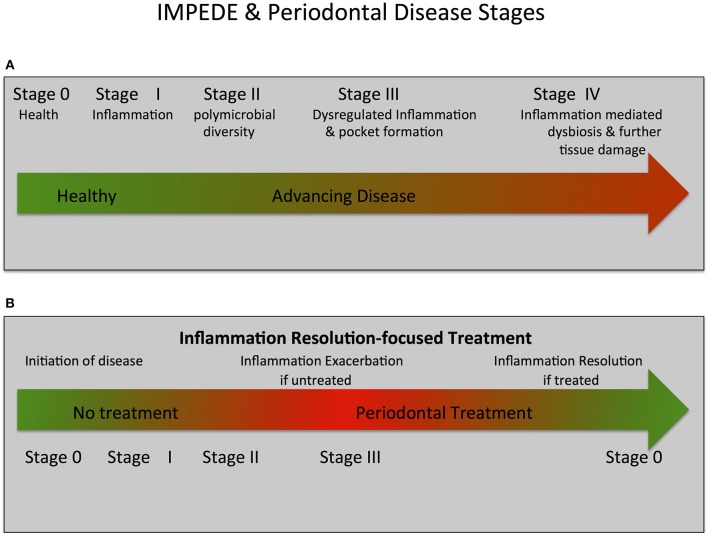
IMPEDE, Periodontal Disease Classification Stages and Treatment.The IMPEDE model proposes that inflammation can manifest for each periodontitis classification stage as a principal driver of the clinical condition. In keeping within the framework of the current classification of periodontitis, the IMPEDE model identifies 5 stages (0–IV) integrating the transition from health, to gingivitis and (if untreated) ultimately to periodontitis. **(A)** IMPEDE stages within the new classification framework. 0 = Periodontal Health; Stage I = Gingivitis (initiation of inflammation); Stage II = Initiation/early periodontitis (Polymicrobial diversity emerges); Stage III = Advancing Periodontitis (dysregulated inflammation and pocket formation) and Stage IV = Late stage periodontitis (Inflammation mediated dysbiosis, opportunistic infection and advanced tissue destruction). **(B)** Inflammation-mediated polymicrobial dysbiosis and tissue damage can be exacerbated if no treatment is provided or can be driven toward resolution of inflammation and tissue repair/regeneration if treatment is provided.

In light of the above assessment of how inflammation mediates dysbiosis and associated exacerbation of periodontal damage, we propose a new unifying hypothesis called the “Inflammation-Mediated Polymicrobial-Emergence and Dysbiotic-Exacerbation” (IMPEDE) Model (Figure 1). This model is designed to complement the current Classification of Periodontal Diseases (39). In this classification, periodontitis is viewed within a continuum from health to disease through 4 stages of severity and complexity as well as extent and distribution. Within the IMPEDE model, we demonstrate how inflammation can manifest within each classification stage as a principal driver of the clinical condition (Figure 2). As for the current classification of periodontitis, the IMPEDE model recognizes 5 stages (0–4) through which health, gingivitis, and periodontitis may develop, be contained or progress. From microbiome analyses and longitudinal clinical studies, there appear to be four different stages of bacterial transitions from health to late stage periodontitis, which is driven by inflammation, pocket formation, and bacterial composition. With health representing Stage 0, there are four subsequent disease development phases: (1) Gingivitis (inflammation associated with an overgrowth of commensal plaque bacteria); (2) Initiation/early periodontitis (Inflammation induced polymicrobial diversity increases and dysbiosis triggered); (3) Inflammation mediated exacerbation of dysbiosis by a self-sustained feedforward loop, and (4) Late stage periodontitis characterized by a decrease in polymicrobial diversity associated with the emergence of a polymicrobial infection.

### Health

Periodontal health has been clearly defined and classified and recognizes the defining feature as being absence of clinical inflammation (39). In addition, it is characterized by a plaque microbiome dominated by commensal gram-positive organisms (e.g., Streptococci, Corynebacteria, Rothia spp.) in homeostasis with the host (20). These species are antagonistic to the gram-negative species (10).

### Gingivitis

Gingival inflammation in response to a non-specific accumulation of dental plaque microbiota is the defining feature of gingivitis (39). This condition is characterized by a chronic overgrowth of largely commensal organisms that in susceptible people causes inflammation and swelling of the soft tissue with early pocket formation. This results in an increase in microbial diversity in the pocket.

### Emergence of Polymicrobial Diversity

It is well-established at oral and other epithelial sites that a buildup of commensal microbial biomass can trigger a switch from homeostasis (tolerance) to inflammation (40). This inflammation produces an increase in diversity of the biofilm, and this can still be associated with health at an epithelial site. Chronic inflammation (gingivitis/moderate periodontitis) can start to change the composition of the plaque with emergence of gram-negative species (21). This diverse polymicrobial biofilm is composed of disease-causing species (pathobionts) and commensal (beneficial) species (symbionts), which can be antagonistic to the pathogens. All these subgingival ecological and environmental changes are driven by the unresolved chronic inflammation, which in turn is intricately related to host susceptibility (genes and environment) and disease (tissue damage and attachment loss). As discussed above, this process can be controlled at this stage by driving resolution of inflammation allowing the commensal biofilm to return as the predominant microbiota.

### Dysbiosis

In susceptible people, the chronic inflammation and anaerobic nature of the pocket results in the emergence and proliferation of certain bacterial species that may be present and an exacerbation of inflammation by the generation of a self-sustained feedforward loop to result in uncontrolled inflammation and tissue destruction (10, 41).

## Opportunistic Polymicrobial Infection

There are many studies that have shown the microbial diversity of subgingival plaque increases from health to disease (gingivitis/periodontitis), which has been attributed to an increase in amount and range of nutrients provided by the exudate associated with chronic gingival inflammation (10, 42). This increase in diversity is distinct from polymicrobial infections at other sites of the body where infection can be characterized by a reduction in diversity due to increased specific pathogen abundance/competition as well as host defense mechanisms decreasing the viability/level of commensal species (43–46). However, most of the studies investigating the microbial diversity at periodontal sites have not differentiated between early/moderate disease and late stage severe periodontitis associated with deep pockets. One study by Kirst et al. (20) did differentiate between 6 mm pockets and those with depths >7–8 mm and they showed that diversity and species richness were significantly higher in 6 mm pockets compared with that of the deeper pockets. The authors also showed that there was a significant increase in abundance of certain species in these deep pockets with Bacteroidetes being the most abundant. In fact, the majority (>50%) of species in the deeper pockets could be defined from families comprising known or suspected periodontal pathogens. This result is similar to those found by other investigators (47, 48) where specific sites associated with severe periodontitis were characterized by an increased abundance of known pathogens. From the Kirst et al. (20) study the authors concluded that subgingival microbial communities at diseased sites were more homogeneous than those at healthy sites suggesting a limited repertoire of species involved in disease progression. These results are consistent with a model of microbial succession in periodontitis in which disease-associated species initially invade/emerge in the healthy microbiota resulting in a diverse community comprising both health and disease-associated species. As disease (attachment loss and pocket depth) progresses, the transitional microbiota is temporally and spatially replaced by predominantly disease-associated species.

Recent studies on the architecture or biogeography of subgingival plaque are consistent with this model as they have revealed considerable spatial and temporal heterogeneity (49–51). These studies have demonstrated considerable site specificity indicating that oral microbes are site/niche specialists and that the fine-scale positioning of a pathogen within a polymicrobial infection site can greatly alter its virulence potential. This site specificity is consistent with the known redox and nutrient gradients evident in periodontal pockets. In this context, it is significant that the periodontal pathogens *Porphyromonas gingivalis* and *Treponema denticola* are predominantly located in deep periodontal pockets at depths of more than 4 mm (52). Furthermore, these pathogens are found together with other periodontal pathogens in microcolony blooms in the biofilm surface layer adjacent to the epithelial lining at the base of deep pockets (52–56). This positioning and co-localization would advantage them in terms of release of outer membrane vesicles loaded with virulence factors into the subjacent tissue and access to the exudate from that inflamed tissue as well as being consistent with the established mutualistic symbiosis and pathogenesis displayed by these species (55, 57–59).

These temporal and spatial analyses of subgingival plaque help explain the disproportional effect these periodontal pathogens can have on dysregulation of the host defense to accelerate attachment loss when they appear to be only a relatively small proportion of the overall microbial biomass removed from a deep periodontal pocket (keystone pathogen hypothesis). Pathogens at the base of a periodontal pocket will be closer and therefore have more influence on subversion of the host immune response and the inflammatory processes resulting in bone resorption occurring under the pocket. These findings also help to explain why the levels of *P. gingivalis* and *T. denticola* above threshold values of around 10–15% of the microbial biomass removed from the pocket could predict imminent attachment loss in a longitudinal prospective clinical trial (60). Hence the levels of periodontal pathogens or “dysbiotic signature” of subgingival plaque may be a useful biomarker/predictor of site disease activity and imminent progression (61).

The dysbiotic signature may not only involve changes in microbial composition, but also changes in metabolomic profile (62, 63). Dysbiosis involves multi-directional metabolic cross-talks between subgingival micro-organisms as well as with the host such that transitions in metabolic profiles may be associated with disease progression. Recently, Sakanaka et al. (64) using saliva metabolomic data demonstrated that the metabolites cadaverine and hydrocinnamate were associated with severe periodontal inflammation. From metabolite set enrichment analysis they showed that polyamine metabolism, arginine and proline metabolism, butyric acid metabolism, and lysine degradation were distinctive metabolic signatures of severe disease.

Taken together, these results indicate a process of inflammation-mediated microbial succession in periodontitis in which disease-associated species temporally and spatially emerge in a periodontal pocket resulting in a dysbiotic state to initiate periodontitis. As disease (attachment loss and pocket depth) progresses the transitional dysbiotic microbiota is further altered by an increase in abundance of predominantly disease-associated species particularly at the base of the periodontal pocket resulting in an opportunistic polymicrobial synergistic infection.

## Concluding Comments

For many years, periodontology has experienced a conundrum with regards to the role of inflammation and microbiology in the pathogenesis of periodontitis. In this paper, we have explored the emerging evidence indicating a convergence of views with the realization of the critical nexus between the microbiology and inflammation. Current evidence suggests that the driver is the inflammation continuum and it is only at a late stage that microbial specificity (pathogenicity) starts to play a role. While this is not a new view, metagenomics, transcriptomics, proteomics, and metabolomics results in animals and humans are producing evidence to support the transition of previous theories into accepted paradigms (38). The emerging concepts presented in this paper are consistent with, and indeed complementary to, the 2017 Classification of Periodontal Diseases (39). The ramifications of these findings now allow focus on how resolving inflammation can lead to a shift in microbial composition and restoration of microbiological balance/homeostasis. In doing so, the nexus between periodontal inflammation and dysbiosis can be harnessed.

## Author Contributions

TV, PB, and ER contributed to conception and design of this review, and critically revised the manuscript.

### Conflict of Interest

The authors declare that the research was conducted in the absence of any commercial or financial relationships that could be construed as a potential conflict of interest.
